# Efficient CRISPR/Cas12a-Based Genome-Editing Toolbox for Metabolic Engineering in *Methanococcus maripaludis*

**DOI:** 10.1021/acssynbio.2c00137

**Published:** 2022-06-22

**Authors:** Jichen Bao, Enrique de Dios Mateos, Silvan Scheller

**Affiliations:** Department of Bioproducts and Biosystems, School of Chemical Engineering, Aalto University, FI-02150 Espoo, Finland

**Keywords:** Methanococcus maripaludis, methanogens, CRISPR/Cas12a, genome editing, metabolic engineering, synthetic biology

## Abstract

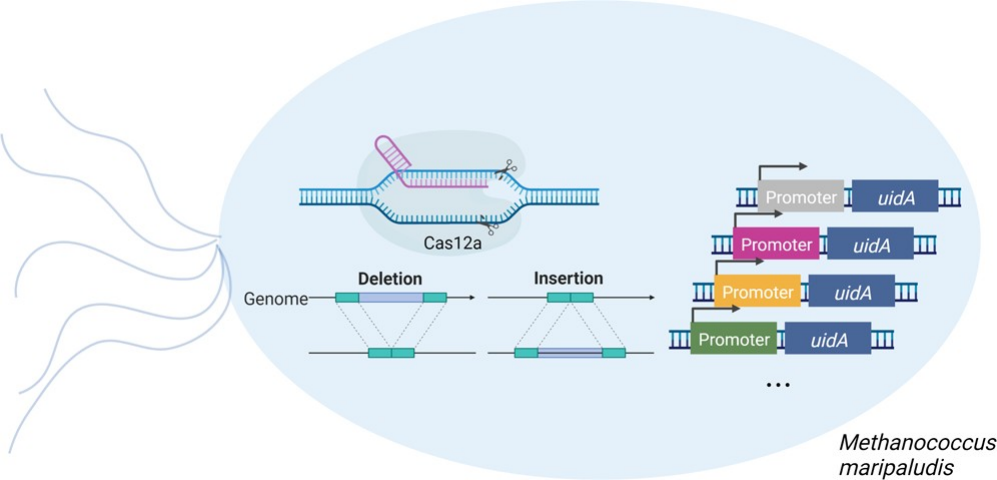

The rapid-growing and genetically tractable methanogen *Methanococcus maripaludis* is a promising host organism for the biotechnological conversion of carbon dioxide and renewable hydrogen to fuels and value-added products. Expansion of its product scope through metabolic engineering necessitates reliable and efficient genetic tools, particularly for genome edits that affect the primary metabolism and cell growth. Here, we have designed a genome-editing toolbox by utilizing Cas12a from *Lachnospiraceae bacterium* ND2006 (LbCas12a) in combination with the homology-directed repair machinery endogenously present in *M. maripaludis*. This toolbox can delete target genes with a success rate of up to 95%, despite the hyperpolyploidy of *M. maripaludis*. For the purpose of demonstrating a large deletion, the *M. maripaludis* flagellum operon (∼8.9 kbp) was replaced by the *Escherichia coli* β-glucuronidase gene. To facilitate metabolic engineering and flux balancing in *M. maripaludis*, the relative strength of 15 different promoters was quantified in the presence of two common growth substrates, either formate or carbon dioxide and hydrogen. This CRISPR/LbCas12a toolbox can be regarded as a reliable and quick method for genome editing in a methanogen.

## Introduction

Methanogenic archaea are biotechnologically employed in a variety of uses, e.g., for methane production in anaerobic digestors,^1^ as biocatalysts in power-to-gas processes,^2^ and as versatile hosts for the development of synthetic pathways that convert carbon dioxide (CO_2_) into value-added products.^3,4^ Hydrogenotrophic methanogens utilize the reductive acetyl-CoA pathway for CO_2_ fixation,^5^ an energy-efficient route to synthesize organic carbon from CO_2_ and hydrogen (H_2_), which is similar to that found in acetogens.^6^ Subtle differences exist between the acetogenic^7^ and methanogenic CO_2_ reduction pathways in terms of ATP investment and cofactor utilization.^8^ Depending on the type of product that needs to be generated from CO_2_ as the carbon source, methanogens may be better-suited hosts than acetogens. Recently, the methanogen *Methanosarcina acetivorans* was re-engineered to no longer depend on methane production for its energy metabolism,^9^ thereby serving as an example where a methanogen could be utilized for generating an expanded repertoire of new potential products besides methane.

*Methanococcus maripaludis* is a promising methanogenic host organism for metabolic engineering of CO_2_-fixation pathways due to its advantageous growth properties, e.g., 2 h doubling time,^10,11^ moderate growth temperature of 38 °C, and ability to fix nitrogen.^12−14^ Typical electron donors for CO_2_ reduction in *M. maripaludis* include formate, H_2_, and bioelectrically coupled systems.^15,16^ Efforts to expand the product scope of *M. maripaludis* beyond methane are already underway. As an example, the mevalonate pathway in this methanogen was metabolically engineered to produce geraniol from CO_2_ and formate.^4^ Efficient and reliable genome-editing tools are critical for successful metabolic engineering in *M. maripaludis*. Marker recycling is a prerequisite for multitarget engineering. In the case of *M. maripaludis*, while a pop-in/pop-out markerless-based genome-editing technique has been developed,^17^ it tends to have a problematic low positive rate, which can sometimes be less than 5%,^17^ particularly for those modifications that affect cell growth. As an alternative, the CRISPR/Cas (clustered regularly interspaced short palindromic repeats/CRISPR associated protein) system might remedy this problem because of its reputation for highly efficient genome editing.

The CRISPR/Cas9 system has already been successfully used for genome editing in a variety of organisms^18−23^ owing to its simplicity and high efficiency, but only a few CRISPR genome-editing toolboxes have been developed for archaea.^24^ The first CRISPR/Cas9-mediated genome-editing system for a methanogen was reported in 2017 using *M. acetivorans* as the model organism.^25^ This Cas9-based system recognizes and cleaves a 20-nucleotide target sequence that is flanked by a 3′-NGG protospacer adjacent motif (PAM). This contrasts with Cas12a, which instead recognizes the 5′-thymine (T)-rich PAM 5′-TTTV. This recognition site makes Cas12a the better option for developing a CRISPR toolbox in microbes with an adenine (A)- and T-rich genome. Another advantageous attribute of Cas12a lies in its ribonuclease activity, which allows the formation of multiple guide RNAs (gRNAs) from a single transcript.^26,27^ Since the *M. maripaludis* genome has a high AT content (67.1%), we decided to use the Cas12a from *Lachnospiraceae bacterium* ND2006 (LbCas12a) and combine it with the intrinsic homology-directed repair machinery to develop a CRISPR genome-editing toolbox. In our study, we examined how the length of the repair fragment (RF) and the distance of the RF to the double-stranded break (DSB) impact on the genome-editing efficiency. As an application of our toolbox, we deleted the *M. maripaludis* flagellum operon and replaced it with the *Escherichia coli* β-glucuronidase gene. To further expand the versatility and editing potential of this genetic toolbox, we also established a Cas9-based editing system, and we quantified the relative strength of 15 different promoters in the presence of two common growth substrates, either formate or H_2_ and CO_2_.

## Results and Discussion

### CRISPR/Cas12a-Based Introduction of Double-Stranded Breaks and Transformation Efficiency

**Figure 1:**
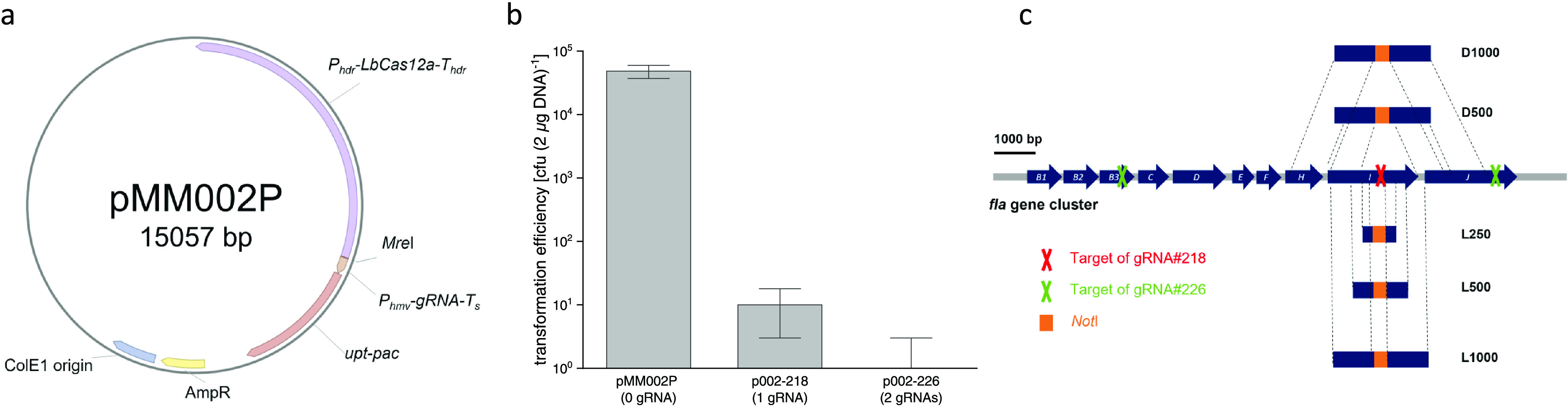
General features of the CRISPR/LbCas12a genome editing. (a) Genetic map of the CRISPR/LbCas12a pMM002P plasmid. The *M. maripaludis* S2 uracil phosphoribosyltransferase gene (*upt*), which serves as a counter-selective marker, and the codon-optimized puromycin *N*-acetyltransferase gene (*pac*) are coexpressed via the *P_mcr_* promoter.^4^ LbCas12a expression is driven by the *P_hdr_* promoter from *Methanococcus voltae* A3. gRNA expression is driven by the *M. voltae* A3 *P_hmv_* histone promoter. Two *PaqC*I sites between the direct repeat sequence and the synthetic terminator in the opposite direction for spacer fusion is used for gRNA insertion (not displayed). The gRNA of the plasmid pMM002P that contains two *PaqC*I sites does not target the chromosome. An *Mre*I restriction site assigned between the gRNA and Cas elements is used for RF insertion. (b) CRISPR/LbCas12a triggered DSBs. Shown are the transformation efficiencies [cfu (2 μg DNA)^−1^] for the CRISPR/LbCas12a pMM002P plasmids with one, two, or no gRNAs that were used to transform *M. maripaludis*. Error bars represent the standard deviation of the values obtained for the transformation efficiency (*n* = 3). (c) Schematic outline of the repair fragment edits. A *Not*I site is placed between the two homologous arms.

For this study, we utilized the host strain *M. maripaludis* JJΔupt in all experiments as well as a recently established natural transformation protocol.^28^ The *E. coli*/*M. maripaludis* shuttle vector pLW40^29^ served as the backbone for constructing the final toolbox plasmid pMM002P (Table S1). Further details about pMM002P and its construction are given in Figure 1a. The transformation efficiency of pMM002P into JJΔupt cells was calculated to be about 5 × 10^4^ colony forming units per 2 μg DNA [cfu (2 μg DNA)^−1^] (Figure 1b), which was similarly obtained for pMM001 (pMM002P lacking LbCas12a) (data not shown). The high transformation efficiency suggests that LbCas12a expression is not toxic in *M. maripaludis*. Because the coexpression of LbCas12a with either one or two gRNA sequences resulted in only 3–18 and 0–3 transformant colonies, respectively (Figure 1b,c), we conclude that the LbCas12a–gRNA complex can cause a lethal DSB in the *M. maripaludis* chromosome. Since nonhomologous end-joining (NHEJ) machineries for DNA repair are rare in archaea,^30^ and as *M. maripaludis* JJ lacks a homolog of the Ku protein (which has a strong binding affinity for free DNA ends or nicks), NHEJ is not expected to provide an escape from such DSBs.

### CRISPR/LbCas12a Genome Editing by Providing a Repair Fragment on the pMM002P-Derived Plasmid

**Figure 2:**
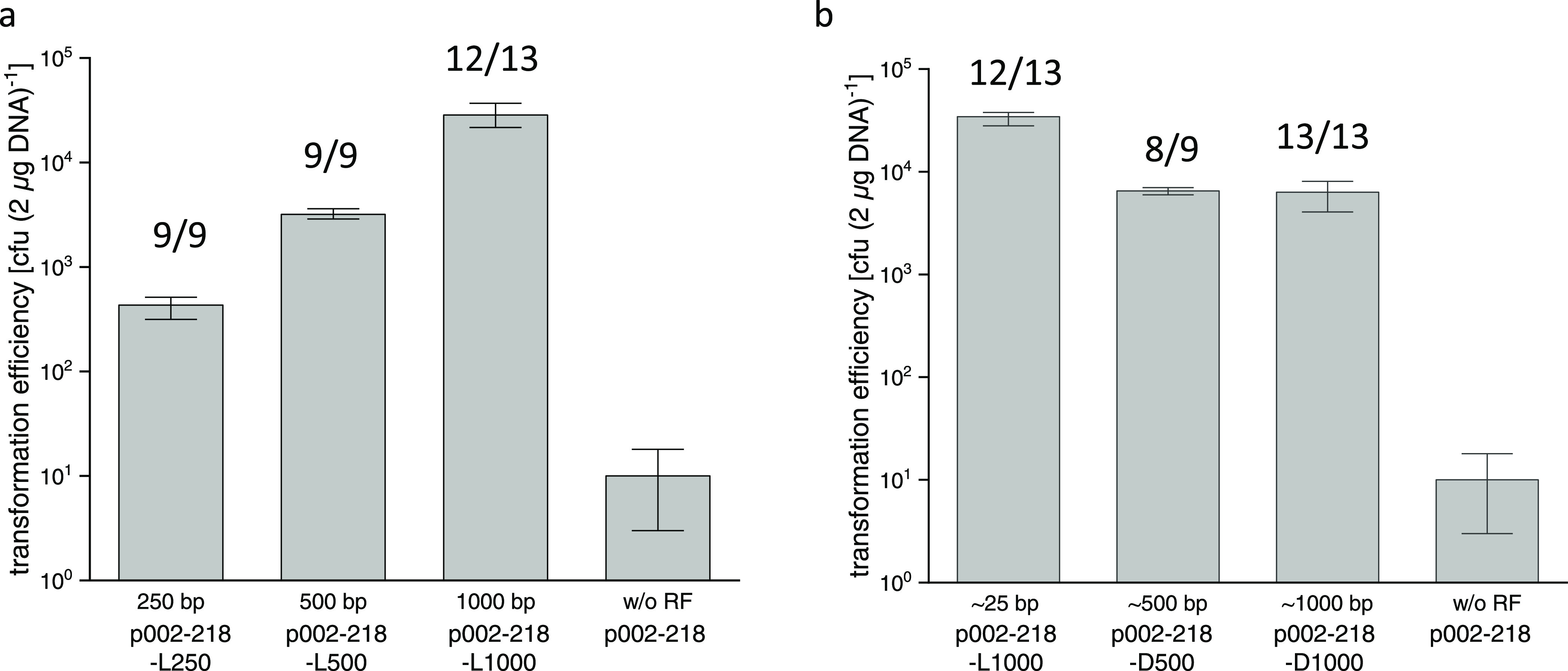
Effect on transformation and genome-editing (positive rates) efficiencies when the length and position of the RF are modified. Transformation efficiency and positive rate in relation to the length and position of the RF. Shown are the transformation efficiencies [cfu (2 μg DNA)^−1^] for the CRISPR/LbCas12a pMM002P-derived plasmids that were used to transform *M. maripaludis*. (a) CRISPR/LbCas12a plasmid p002-218, in which the lengths of the homology arms flanking the RF are 250, 500, and 1000 bp (p002-218-L250, p002-218-L500, and p002-218-L1000, respectively). The distance from the RF to DSB for all plasmids is ∼25 bp. (b) CRISPR/LbCas12a plasmid p002-218 with 1000 bp homologous arms, in which the distance between the RF and the DSB is ∼25, ∼500, and ∼1000 bp (p002-218-L1000, p002-218-D500, and p002-218-D1000, respectively). p002-218 without the RF is included as a control. Error bars represent the standard deviation of the values obtained for the transformation efficiency (*n* = 3). Positive rates representing the fraction of correctly edited colonies per colonies tested by PCR are shown for all plasmid transformations (numbers above bars).

For CRISPR/LbCas12a genome editing, a gRNA was expressed on pMM002P (p002-218) that targets the *flaI* (*MMJJ_11570*) gene of the *M. maripaludis* flagellum operon. The lethal effect of the functional gRNA could be relieved by including RFs with various lengths of the homology arms (Figures 1c and 2a). A 1000 bp homology arm on either side resulted in a transformation efficiency of 2.8 × 10^4^ cfu (2 μg DNA)^−1^. We therefore used homology arms of this length for all subsequent experiments. While 250 bp homology arms were long enough to repair DSBs created by the Cas12a/gRNA complex, the transformation efficiency was 70 times lower. We also examined what effect the distance between the RF and the DSB would have on the transformation efficiency by testing three different distances (i.e., ∼25, ∼500, and ∼1000 bp) (Figures 1c and 2b). The transformation efficiency was found to be five times lower with the 500 bp distance than with the 25 bp distance (two-sided *t*-test, *P* < 0.001), but no significant difference was observed whether 500 or 1000 bp distances were used (two-sided *t*-test, *P* > 0.05). Because *M. maripaludis* contains an active *Pst*I restriction modification system, cells are able to digest foreign DNA containing unmethylated *Pst*I sites, which lowers the transformation efficiency by 1.6–3.4 fold per *Pst*I site.^31^ This reduction of the transformation efficiency is exemplified by the presence of one *Pst*I site in each of the 500 and 1000 bp homology arms, as any restriction digestion would likely be responsible for a lower number of transformants (Figure 2b). The similar transformation efficiency obtained with the 500 and 1000 bp distances to the DSB suggests that the genome-editing efficiency is unaffected within a distance length of 1000 bp. To assess the positive rate of genome editing, two sets of primers were used to amplify a DNA sequence on both sides of the homology arms on the chromosome. Each of the PCR products was then subjected to *Not*I digestion. Here, a *Not*I restriction site was engineered between the left and right RFs, so that the wild-type polyploid genome copies are distinguishable from the edited ones (Figure 1c). The PCR products were sequenced afterward to confirm that the sites were edited as expected. As a result, genome editing was highly efficient, displaying a positive rate of 89–100% (Figure 2a). All results taken together (see Figure 2), 63 out of 66 colonies had been correctly edited, which equates to an average positive rate of nearly 95%. Hence, we conclude that our CRISPR/LbCas12a toolbox can reliably perform genome editing in *M. maripaludis* (see the Supporting information for a detailed description of the general procedure for utilizing the CRISPR/LbCas12a genome-editing toolbox in *M. maripaludis*).

### CRISPR/LbCas12a Genome Editing by Providing a Repair Fragment Separately

To help speed up the construction of different genome-edited mutants in parallel, we modified our CRISPR/LbCas12a toolbox by providing the RF separately as a suicide plasmid. For this alternative procedure, the CRISPR/LbCas12a cleavage plasmid was cotransformed with a suicide plasmid containing a promoter–*uidA* fusion expression cassette (for further details, see the section below on promoter strengths) flanked on either side by 1000 bp homology arms. While we successfully obtained transformants with this modification, the transformation efficiency was lowered by 10–50 fold, but the genome-editing efficiency was robust and remained high. As proof, when we randomly selected and examined three transformants from the 15 different genome-edited constructs made using the suicide plasmid, all of them (45/45) were found to be positive (data not shown). We also examined the possibility of cotransforming the CRISPR/LbCas12a cleavage plasmid with the RF separately by providing it as a linear PCR product. In this case, while genome editing was deemed successful, the transformation efficiency was 100–1000 times lower than that obtained using our original CRISPR/LbCas12a toolbox method with the plasmid containing the 1000 bp homology arm (p002-218-L1000).

### Using CRISPR/LbCas12a Genome Editing to Replace a Large Genome Fragment with a Heterologous Gene

**Figure 3:**
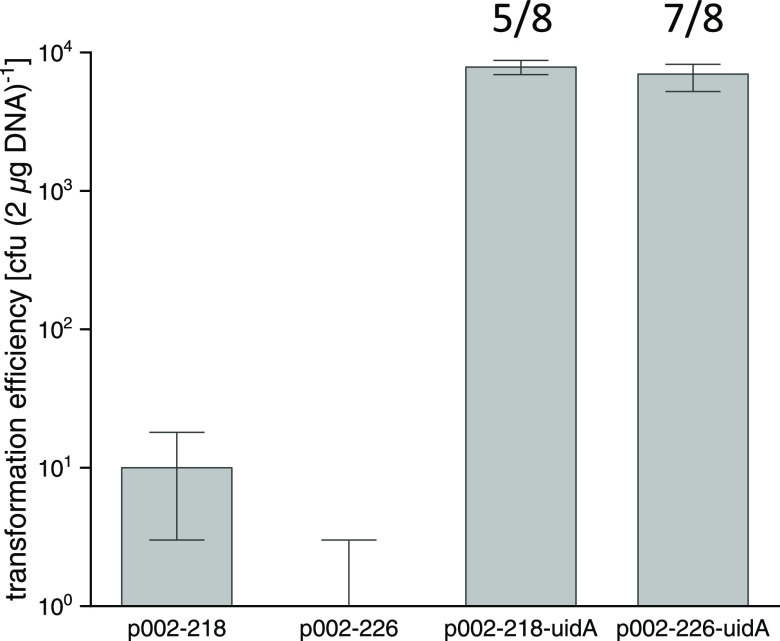
CRISPR/LbCas12a genome-edited replacement of the *M. maripaludis* flagellum operon with the *E. coli* β-glucuronidase gene (*uidA*). Shown are the transformation efficiencies [cfu (2 μg DNA)^−1^] for the CRISPR/LbCas12a pMM002P-derived plasmids that were used to transform *M. maripaludis*. Plasmids p002-218 and p002-226 (controls) express one and two gRNAs, respectively, and do not contain the RF. Plasmids p002-218-uidA and p002-226-uidA express one and two gRNAs, respectively, but contain the RF. Error bars represent the standard deviation of the values obtained for the transformation efficiency (*n* = 3). Positive rates representing the fraction of correctly edited colonies per colonies tested by PCR are shown for the p002-218-uidA and p002-226-uidA transformations (numbers above bars).

As an application of our CRISPR/LbCas12a method, we demonstrated its use as a tool for heterologous gene integration. We removed the entire ∼8.9 kbp flagellum operon (*flaB1B2B3CDEFGHIJ MMJJ_11660 – MMJJ_11560*) from the *M. maripaludis* chromosome and substituted it with the *E. coli* β-glucuronidase gene (*uidA*). This edit was performed using two different CRISPR/LbCas12a plasmids: p002-218-uidA, which has one gRNA that generates a single lethal DSB on the chromosome, resulting in two long distances between the DSB and the RF (i.e., 6.4 and 2.5 kbp), and p002-226-uidA, which has two different gRNAs that cleave at either side of the flagellum operon and thus shorten the distances between the DSB and the RF (i.e., 0.25 and 1.6 kbp). Both plasmids resulted in a similar transformation efficiency (Figure 3), demonstrating that our CRISPR/LbCas12a method can successfully generate a large chromosomal fragment deletion. The positive rate of genome editing was lower when only one gRNA was used, as it seems the 6.4 kbp distance (Figure 3) affects the positive rate by exceeding the 1000 bp length. A similar effect was observed for *M. acetivorans*, in which the positive rate was significantly reduced when the distance to the DSB went beyond 1000 bp.^25^ In addition, the transformation efficiency had also decreased with an increasing distance.^25^ Using two gRNAs to shorten the distance between the RF and the DSB might help improve the transformation and genome-editing efficiencies also in other methanogen-based CRISPR systems.

To be ready for a second round of genome editing in the future, the CRISPR plasmid was removed after genome editing by being counter-selected on a plate containing 6-azauracil. The absence of the plasmid was confirmed by the inability of the cells to grow on the medium containing puromycin, and the removal rate was 9/10.

### Development of the CRISPR/SpCas9 Genome-Editing System

**Figure 4:**
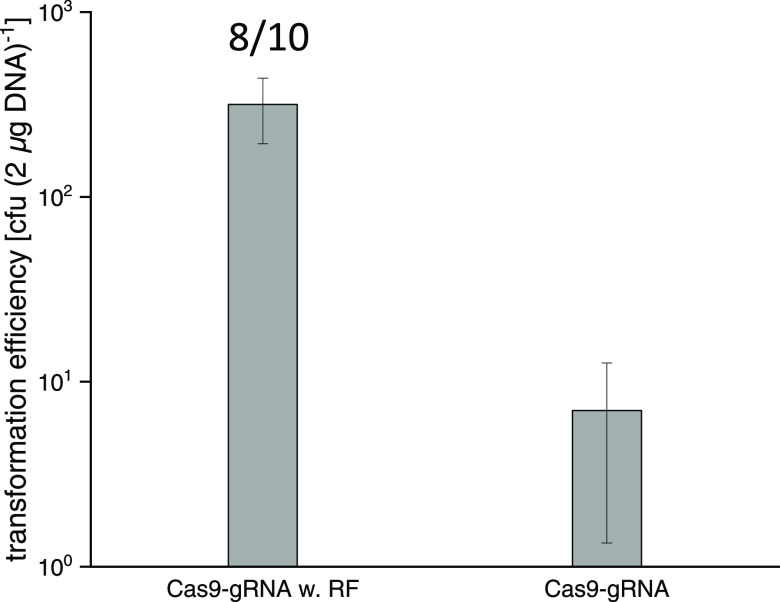
CRISPR/SpCas9 genome-edited replacement of the *M. maripaludis* alanine dehydrogenase–alanine racemase (*ald-alr* 1.9 kbp) genes with a 4.2 kbp fragment. Shown are the transformation efficiencies [cfu (2 μg DNA)^−1^] for the CRISPR/SpCas9 plasmids that were used to transform *M. maripaludis*. The left bar indicates that the cells were transformed with a suicide plasmid containing the integration cassette and a CRISPR/SpCas9 plasmid carrying a gRNA targeting to the *ald-alr*. The right bar indicates that the cells were only transformed with a CRISPR/SpCas9 plasmid carrying a gRNA targeting to the *ald-alr*. Error bars represent the standard deviation of the values obtained for the transformation efficiency (*n* = 3). The positive rate representing the fraction of correctly edited colonies per colonies tested by PCR is shown for the transformations (numbers above the left bar).

To expand the gRNA repertoire in our CRISPR/Cas toolbox, we also constructed a Cas9-based genome-editing plasmid that uses the *Streptococcus pyogenes* Cas9 endonuclease. With this CRISPR/SpCas9 tool, we were able to successfully replace the 1.9 kbp fragment covering the alanine dehydrogenase–alanine racemase genes (*ald*-*alr, MMJJ_13250 – MMJJ_13260*) in the *M. maripaludis* chromosome with a 4.2 kbp DNA fragment containing a different heterologous gene. Here, the CRISPR/SpCas9 cleavage plasmid with one gRNA was cotransformed with a suicide plasmid containing the 4.2 kbp DNA fragment flanked on both sides by 1000 bp homology arms. The transformation efficiency was only 317 ± 123 cfu (2 μg DNA)^−1^, but the rate of genome editing was high (8/10 colonies were edited) (Figure 4). Since the RF contained two *Pst*I sites, they can explain the lower transformation efficiency of this genome-editing system.

### Promoter Strengths of 15 Different Promoter–*uidA* Fusions Constructed by CRISPR/LbCas12a

**Figure 5:**
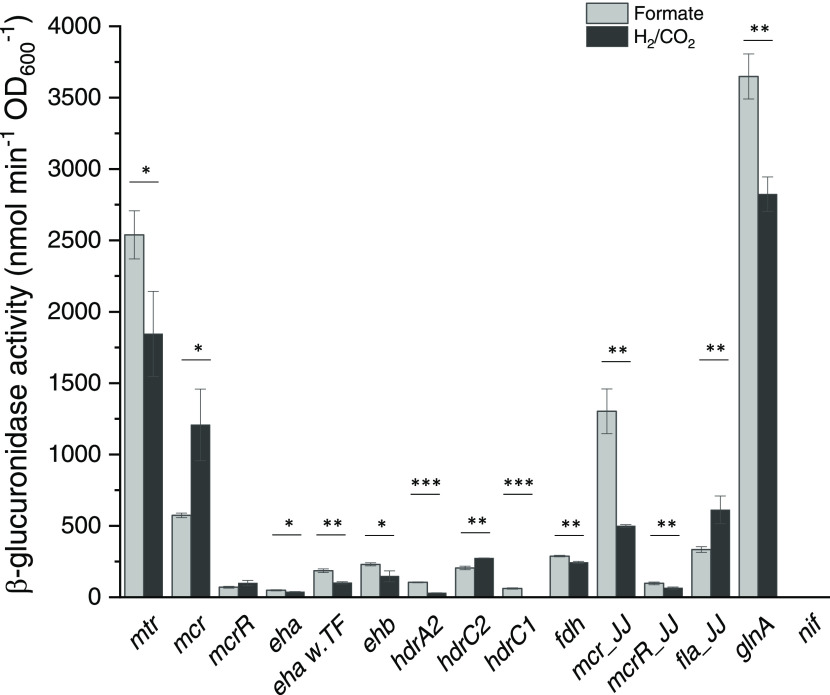
Quantification of promoter strengths for the two different growth conditions formate or H_2_/CO_2_, measured after the culture has reached OD_600_ = ca. 0.5. The promoters *mcr_JJ*, *mcrR_JJ*, and *fla_JJ* are from *M. maripaludis* JJ. The remaining promoters are from *M. vannielii* SB. Error bars represent the standard deviation (*n* = 3). The activity of the *hdrC1* promoter in H_2_/CO_2_ medium and the *nif* promoter in formate and H_2_/CO_2_ medium cannot be detected. *P < 0.05; ^**^P < 0.01; ^***^P < 0.001.

Fifteen different promoters delivered as a promoter–*uidA* fusion expression cassette were integrated into the locus of the acetyl-CoA synthetase gene (*MMJJ_0*9*370*) of *M. maripaludis* JJ using the CRISPR/LbCas12a toolbox (for further details, see earlier section; the sequences of the 15 promoters are listed in the Supporting information). Three promoters (*P*_*mcr*_JJ_, *P*_*mcrR*_JJ_, and *P*_*fla*_JJ_) originated from *M. maripaludis* JJ, while the remaining twelve were from the closely related methanogen, *Methanococcus vannielii* SB. The relative strengths of these promoters were measured in the presence of two common growth substrates, either formate or H_2_ and CO_2_. All promoters except *P*_*nif*_ and *P*_*hdrC1*_ had successfully driven the expression of *uidA* using both growth substrates (Figure 5). *P*_*hdrC1*_ had allowed gene expression in only the formate-containing growth medium, while no expression was detected for *P*_*nif*_ in both growth substrates. Since *P*_*mcr*_ is regarded as a strong constitutive promoter in methanogens,^32^ then by comparison, *P*_*glnA*_, *P*_*mtr*_, *P*_*mcr*_, *P*_*mcr*_JJ_, and *P*_*fla*_JJ_ can be judged as strong promoters in *M. maripaludis*. Transcription from *P*_*nif*_ is normally repressed by the nitrogen regulatory protein R (NrpR) but can become highly active when N_2_ gas serves as the sole nitrogen source or else in the absence of NrpR.^33^ With this in mind, we deleted the *nrpR* gene from the genome-edited *P*_*nif*_*–uidA* strain and found that *P*_*nif*_ was no longer repressed and had instead increased in strength significantly (2670 ± 58 nmol min^–1^ OD_600_^–1^) (two-sided *t*-test, *P* < 0.001). It is tempting to speculate that the Δ*nrpR*–*P*_*nif*_ strain might be a useful host for target genes requiring very strong expression. For the majority of the promoters, their strengths were similar for both growth substrates.

While *P*_*eha*_ normally drives the expression of the energy-converting hydrogenase A gene (*eha*) in *M. vannielii*, it was observed as a weak promoter in *M. maripaludis*. *P*_*eha*_ does not directly control the *eha* gene expression, but instead first drives the expression of a putative transcriptional factor (TF) gene that precedes the *eha* gene, wherein both genes are presumably part of an operon. We also examined a *uidA* construct that includes the TF gene after the *P*_*eha w.TF*_ promoter sequence. The *uidA* expression using both growth substrates was found to be significantly higher for the *P*_*eha w.TF*_ construct than the *P*_*eha*_ one (two-sided *t*-test, *P* < 0.01). These results suggest that this transcriptional factor might regulate *eha* expression. On the other hand, *P*_*glnA*_ from *M. vannielii* was unexpectedly strong in *M. maripaludis*, even with the presence of the NrpR repressor or ammonium in the growth medium. DNA sequencing of the integrated *P*_*glnA*_*–uidA* expression cassette eliminated a possible point mutation or other change as being responsible for this unusual promoter strength. Likewise, constructing and testing a new *P*_*glnA*_–*uidAM. maripaludis* strain still gave the same result. Thus, one can confidently conclude that *P*_*glnA*_ from *M. vannielii* functions as a strong promoter in *M. maripaludis*. In *M. maripaludis*, native *P*_*glnA*_ normally directs basal constitutive expression levels when ammonium is present.^34^ Although the *glnA* operator for *P*_*glnA*_ is the same in *M. vannielii* and *M. maripaludis*, the *P*_*glnA*_*–uidA* strain appears to have the highest promoter strength among all others tested in this study.

## Conclusions

*M. maripaludis* already possesses several efficient genetic tools and transformation protocols for standard applications,^17,28,31^ e.g., such as the classic pop-in/pop-out genome-editing technique.^17^ In some instances, however, this genetic editing tool is hindered by a low positive rate, with many colonies requiring to be screened to obtain a desired genotype, particularly when the targets to be engineered affect cell growth.^32^ As a solution, we have developed a reliable CRISPR/Cas12a toolbox that can efficiently knock-in or knock-out genes in *M. maripaludis* with a positive rate of at least 95%. Notably, our system requires only a single round of homologous recombination and lacks merodiploid formation, which then lowers the workload of genome editing and increases the overall success rate. The option of providing the RF separately as a suicide plasmid or PCR fragment might further speed up the genome-editing process. Since Cas12a displays ribonuclease activity that can process a single continuous multi-gRNA transcript,^26,27^ it might be convenient to express two gRNAs via our CRISPR/LbCas12a system, thus saving the time and cost of additional plasmid construction. Our CRISPR/LbCas12a toolbox can also allow for heterologous protein production in *M. maripaludis*, as it drives the stable integration of genes into the chromosome. *M. maripaludis* might become an attractive expression host for many proteins that are difficult to be produced in *E. coli*, e.g., such as formate dehydrogenase,^16^ methyl-coenzyme M reductase,^35^ and heterodisulfide reductase.^36^ While a variety of promoters have thus far been studied and used for synthetic biology in *M. maripaludis,*(14,33,37,38) a uniform system to compare their strengths has been lacking. In that context, our CRISPR/LbCas12a genome-editing toolbox now represents a versatile system for engineering and balancing metabolic fluxes in *M. maripaludis* strains.

## Materials and Methods

### Plasmids and Strains

All plasmids and strains used in this study are listed in Tables S1 and S2, respectively. Links to plasmid maps are listed in Table S3. *M. maripaludis* JJΔupt^28^ and plasmid pLW40^29^ are gifts from Prof. Kyle Costa, University of Minnesota. Plasmid pMEV4^4^ was kindly provided by Prof. William B Whitman, University of Georgia. *M. maripaludis* S2^39^ was kindly provided by Prof. John Leigh and Dr. Thomas Lie, University of Washington. *E. coli* NEB5α (New England Biolabs) was used for plasmid construction. The plasmids pMM002P and pMM005 were constructed by Gibson assembly.^40^ The construction protocol and primers for pMM002P and pMM005 are described in Tables S4 and S5, respectively. All of the cleavage plasmids were constructed in the following manner. For LbCas12a gRNA, the forward primer consisted of 5′-AGAT and 24-nucleotide guide sequence, whereas the reverse primer consisted of 5′-TATC and 24-nucleotide reverse complement guide sequence. For SpCas9 gRNA, the forward primer consisted of 5′-AGTG and 20-nucleotide guide sequence, whereas the reverse primer consisted of 5′-AAAC and 20-nucleotide reverse complement guide sequence. Both sets of forward and reverse primers containing the gRNA and a 5′ four-nucleotide overhang were annealed. The annealing product was ligated to *PaqC*I-digested pMM002P or pMM005 vector DNA. CRISPR guide sequences were designed using the CHOPCHOP webtool (https://chopchop.cbu.uib.no/).^41^ The RF was inserted into the corresponding cleavage plasmid at the *Mre*I restriction site. Additional primers used in this study are listed in Table S6.

### Growth Media and Conditions

Lysogeny broth medium (10 g L^–1^ tryptone, 10 g L^–1^ NaCl, and 5 g L^–1^ yeast extract) containing 50 mg L^–1^ ampicillin was used for plasmid construction. Liquid McC medium was used for growing *M. maripaludis* strains with an anoxic headspace (2.8 bar, 80% H_2_/20% CO_2_).^28^ Sealed culture tubes were incubated at 37 °C with 200 rpm agitation. McFC medium with an anoxic headspace (1 bar, 80% N_2_/20% CO_2_) was used when formate served as the carbon source.^42^ Sealed culture tubes were incubated statically at 37 °C. Puromycin (2.5 μg mL^–1^) or 6-azauracil (0.25 mg mL^–1^) was added as required.

### *M. Maripaludis* Transformation

The natural transformation of *M. maripaludis* was performed using a previously described protocol.^28^ Briefly, a sealed tube containing a 5 mL of *M. maripaludis* culture was grown overnight to an OD_600_ between 0.7 and 1.2. Two micrograms of DNA was then added directly to the culture. This was followed by flushing the headspace with a gas mixture of 80% H_2_ and 20% CO_2_ for 30 s and adjusting the pressure to 2.8 bar. The sealed culture tube was then incubated at 37 °C with 200 rpm agitation for 4 h, after which the cells were spread-plated onto solid McC medium supplemented with 2.5 μg mL^–1^ puromycin and grown anaerobically at 37 °C.

### Curing of the CRISPR/Cas Plasmid from *M. Maripaludis* Strains

*M. maripaludis* strains containing the CRISPR/Cas toolbox plasmid were grown in 5 mL of liquid McC medium without antibiotics to an OD_600_ between 0.7 and 1.2. A 100 μL aliquot of each culture was used to inoculate another 5 mL of liquid McC medium lacking antibiotics and allowed to incubate overnight. A single droplet of culture was then streaked out onto solid McC medium containing 0.25 mg mL^–1^ 6-azauracil. After 3–5 days, several isolated colonies were selected and streaked out onto another plate of the same medium for purification of plasmid-free cells.

### β-Glucuronidase Activity Measurements

4-Nitrophenyl β-d-glucuronide (4-NPG, Sigma-Aldrich) served as the substrate and was prepared as a 10 mg mL^–1^ stock solution in 50 mM sodium phosphate buffer, pH 7.0 (Na-PB). For measuring β-glucuronidase activity, a tube of *M. maripaludis* cells was first grown to an OD_600_ of ∼0.5 (BioPhotometer plus, Eppendorf) and then a 1 mL aliquot of culture was centrifuged at 10 000 *g* for 2 min. The pelleted cells were resuspended with Na-PB (500 μL) and the cell suspension was subjected to glass bead (30 μL) disruption for 5 min. Afterward, the cell-free lysate was recovered by centrifugation at 10 000 *g* for 2 min. To take activity measurements, the cell-free lysate was diluted appropriately to 500 μL of Na-PB and incubated for 20 min at 37 °C. A 40 μL aliquot of 4-NPG stock solution (see above) was added to the mix and allowed to react for 15 min at 37 °C. The reaction was stopped by adding a 400 μL aliquot of 200 mM sodium carbonate, and the absorbance measurement was taken at 405 nm with a UV–vis spectrophotometer. Specific activity calculations were made with *E. coli* K12 β-glucuronidase (Cat. no. 3 707 580 001, Sigma-Aldrich) as the standard using the conversion factor of 398 nmol min^–1^.

## Supporting Information Available

Plasmids; strains and primers lists; plasmid maps; guidelines on the use of the CRISPR/Cas genome-editing toolbox in *M. maripaludis;**M. maripaludis* natural transformation protocol; and promoter sequences (PDF)
