# Supplementary material for: Efficient CRISPR/Cas12a-Based Genome-Editing Toolbox for Metabolic Engineering in Methanococcus maripaludis
Source: ACS Synth Biol. 2022 Jun 22;11(7):2496–503. doi: 10.1021/acssynbio.2c00137 (PMC9295151; doi:10.1021/acssynbio.2c00137)
Supplement: Supplementary file 1 — sb2c00137_si_001.pdf [file sb2c00137_si_001.pdf]

Supporting information for article:

Efficient CRISPR/Cas12a-based genome editing toolbox for metabolic engineering in *Methanococcus maripaludis*

Jichen Bao, Enrique de Dios Mateos, Silvan Scheller*

Department of Bioproducts and Biosystems, School of Chemical Engineering, Aalto University, FI-02150, Espoo, Finland

*corresponding author: Silvan Scheller (silvan.scheller@aalto.fi)

Table S1. Plasmid list

Plasmid	Description	Reference
pLW40	*E. coli*/*M. maripaludis* shuttle vector; the backbone of pMM002P	[1]
pMEV4	*E. coli*/*M. maripaludis* shuttle vector, only replicates in *M. maripaludis* S0001[2], carrying codon-optimized *pac*	[3]
pMEV4mTs	Derived from pMEV4 carrying the synthetic terminator (*Ts*)	Provided by Prof. Whitman
pY016	Nucleic acid sequence source of LbCas12a	[4]
pX165	Nucleic acid sequence source of SpCas9	[5]
pMM002P	CRISPR/LbCas12a toolbox plasmid	This study
pMM005	CRISPR/SpCas9 toolbox plasmid	This study
p002-218	pMM002P containing a guide sequence 5'-CCCACAGATGAATATACTCATTCC targeting to *flaI* (*MMJJ_11570*)	This study
p002-226	pMM002P containing guide sequences 5'-GATATACCCGACACAATGATATCG and 5' -GTGTAACCATAACTGCTAATTTAT targeting to *flaJ* (*MMJJ_11560*) and *flaB3* (*MMJJ_11640*)	This study
p002-218-L1000	p002-218, 1000 bp long homologous arms on each side, 25 bp from the RF to DSB	This study
p002-218-L500	p002-218, 500 bp long homologous arms on each side, 25 bp from the RF to DSB	This study
p002-218-L250	p002-218, 250 bp long homologous arms on each side, 25 bp from the RF to DSB	This study
p002-218-D500	p002-218, 1000 bp long homologous arms on each side, 500 bp from the RF to DSB	This study
p002-218-D1000	p002-218, 1000 bp long homologous arms on each side, 1000 bp from the RF to DSB	This study
p002-218-uidA	p002-218, P_{fla_JJ}-*uidA*-T_{fla}; the 1000 bp homologous arms on each side covers the promoter and the terminator of the flagellum operon	This study
p002-226-uidA	p002-226, P_{fla_JJ}-*uidA*-T_{fla}; the 1000 bp homologous arms on each side covers the promoter and the terminator of the flagellum operon	This study
p002-253	pMM002P containing a guide sequence 5'-AATGCAAGCTACAACTGTGTAGAC targeting to *acs* (*MMJJ_09370*)	This study
p002-247	pMM002P containing a guide sequence 5'-AGTGATACCTAACAGCCCTTTCTC targeting to *nrpR* (*MMJJ_03770*)	This study
pPmtr-uidA	suicide plasmid containing "P_{mtr}-*uidA*" cassette, 1000 bp long homologous arms on each side, the promoter is from *M. vannielli* SB	This study
pPmcr-uidA	suicide plasmid containing "P_{mcr}-*uidA*" cassette, 1000 bp long homologous arms on each side, the promoter is from *M. vannielli* SB	This study
pPmcrR-uidA	suicide plasmid containing "P_{mcrR}-*uidA*" cassette, 1000 bp long homologous arms on each side, the promoter is from *M. vannielli* SB	This study

pPhdrC1-uidA	suicide plasmid containing "*P_{hdrC1}-uidA*" cassette, 1000 bp long homologous arms on each side, the promoter is from *M. vannielli* SB	This study
pPhdrC2-uidA	suicide plasmid containing "*P_{hdrC2}-uidA*" cassette, 1000 bp long homologous arms on each side, the promoter is from *M. vannielli* SB	This study
pPhdrA2-uidA	suicide plasmid containing "*P_{hdrA2}-uidA*" cassette, 1000 bp long homologous arms on each side, the promoter is from *M. vannielli* SB	This study
pPfdh-uidA	suicide plasmid containing "*P_{fdh}-uidA*" cassette, 1000 bp long homologous arms on each side, the promoter is from *M. vannielli* SB	This study
pPeha-uidA	suicide plasmid containing "*P_{eha}-uidA*" cassette, 1000 bp long homologous arms on each side, the promoter is from *M. vannielli* SB	This study
pPeha w.TF-uidA	suicide plasmid containing "*$P_{eha\ w.TF}$-uidA*" cassette, 1000 bp long homologous arms on each side, the promoter is from *M. vannielli* SB	This study
pPehb-uidA	suicide plasmid containing "*P_{ehb}-uidA*" cassette, 1000 bp long homologous arms on each side, the promoter is from *M. vannielli* SB	This study
pPglnA-uidA	suicide plasmid containing "*P_{glnA}-uidA*" cassette, 1000 bp long homologous arms on each side, the promoter is from *M. vannielli* SB	This study
pPnif-uidA	suicide plasmid containing "*P_{nif}-uidA*" cassette, 1000 bp long homologous arms on each side, the promoter is from *M. vannielli* SB	This study
pPmcr_JJ-uidA	suicide plasmid containing "*P_{mcr_JJ}-uidA*" cassette, 1000 bp long homologous arms on each side, the promoter is from *M. maripaludis JJ*	This study
pPmcrR_JJ-uidA	suicide plasmid containing "*P_{mcrR_JJ}-uidA*" cassette, 1000 bp long homologous arms on each side, the promoter is from *M. maripaludis JJ*	This study
pPfla_JJ-uidA	suicide plasmid containing "*P_{fla_JJ}-uidA*" cassette, 1000 bp long homologous arms on each side, the promoter is from *M. maripaludis JJ*	This study
pΔnrpR	suicide plasmid containing deletion cassette of *nrpR*, a P_{mcr} promoter was placed between the homologous arms to drive the expression of *rlmE*, which is at the downstream of *nrpR*	This study
p005-380	pMM005 containing guide sequences 5'-TATTGAGATGTGTTTCCGGG targeting to *alr* (*MMJJ_13260*)	This study
p4.2k	suicide plasmid containing a 4.2 kb insertion flanking with ca. 1000 bp upstream of *ald* (*MMJJ_13250*) and 1000 bp downstream of *alr* (*MMJJ_13260*)	This study

Table S2. Strain list

Strain	Genotype	Remarks	Reference
S2	wildtype	Used as PCR template for *upt* gene	[6]
SB	*Methanococcus vannielii*	Used as PCR template for promoters	Purchased from DSMZ
A3	*Methanococcus voltae*	Used as PCR template for promoter and terminator for Cas protein expression	Provided by Prof. Whitman
JJΔupt	Δ*upt*		[7]
JL1000	Δ*upt* Δ*flaI(849-904)::gcggccgc* with p002-218-L1000	JJΔupt was transformed with p002-218-L1000	This study
JL500	Δ*upt* Δ*flaI(849-904)::gcggccgc* with p002-218-L500	JJΔupt was transformed with p002-218-L500	This study
JL250	Δ*upt* Δ*flaI(849-904)::gcggccgc* with p002-218-L250	JJΔupt was transformed with p002-218-L250	This study
JD500	Δ*upt* Δ*flaI(378-1379)::gcggccgc* with p002-218-D500	JJΔupt was transformed with p002-218-D500	This study
JD1000	Δ*upt* Δ*flaH* (*MMJJ_11580*)*(621-693)IJ(1-179)::gcggccgc* with p002-218-D1000	JJΔupt was transformed with p002-218-D1000	This study
J218U	Δ*upt* Δ*flaB1B2B3CDEFGHIJ* (*MMJJ_11660 - MMJJ_11560*)*::uidA*	JJΔupt was transformed with p002-218-uidA, plasmid was removed afterwards.	This study
J226U	Δ*upt* Δ*flaB1B2B3CDEFGHIJ::uidA*	JJΔupt was transformed with p002-226-uidA, plasmid was removed afterwards.	This study
Jmtr	Δ*upt* Δ*acs(107-1075)::*P_{mtr}*-uidA*	JJΔupt was transformed with p002-253 and pPmtr-uidA, plasmid was removed afterwards.	This study
Jmcr	Δ*upt* Δ*acs(107-1075)::*P_{mcr}*-uidA*	JJΔupt was transformed with p002-253 and pPmcr-uidA, plasmid was removed afterwards.	This study
JmcrR	Δ*upt* Δ*acs(107-1075)::*P_{mcrR}*-uidA*	JJΔupt was transformed with p002-253 and pPmcrR-uidA, plasmid was removed afterwards.	This study
JhdrC1	Δ*upt* Δ*acs(107-1075)::*P_{hdrC1}*-uidA*	JJΔupt was transformed with p002-253 and pPhdrC1-uidA, plasmid was removed afterwards.	This study
JhdrC2	Δ*upt* Δ*acs(107-1075)::*P_{hdrC2}*-uidA*	JJΔupt was transformed with p002-253 and pPhdrC2-uidA, plasmid was removed afterwards.	This study

JhdrA2	*Δupt Δacs(107-1075)::*P_{hdrA2}*-uidA*	JJΔupt was transformed with p002-253 and pPhdrA2-uidA, plasmid was removed afterwards.	This study
Jfdh	*Δupt Δacs(107-1075)::*P_{fdh}*-uidA*	JJΔupt was transformed with p002-253 and pPfdh-uidA, plasmid was removed afterwards.	This study
Jeha	*Δupt Δacs(107-1075)::*P_{eha}*-uidA*	JJΔupt was transformed with p002-253 and pPeha-uidA, plasmid was removed afterwards.	This study
Jehawtf	*Δupt Δacs(107-1075)::*$P_{eha\ w.TF}$*-uidA*	JJΔupt was transformed with p002-253 and pPeha w.TF-uidA, plasmid was removed afterwards.	This study
Jehb	*Δupt Δacs(107-1075)::*P_{ehb}*-uidA*	JJΔupt was transformed with p002-253 and pPehb-uidA, plasmid was removed afterwards.	This study
JglnA	*Δupt Δacs(107-1075)::*P_{glnA}*-uidA*	JJΔupt was transformed with p002-253 and pPglnA-uidA, plasmid was removed afterwards.	This study
Jnif	*Δupt Δacs(107-1075)::*P_{nif}*-uidA*	JJΔupt was transformed with p002-253 and pPnif-uidA, plasmid was removed afterwards.	This study
JmcrJ	*Δupt Δacs(107-1075)::*P_{mcr_JJ}*-uidA*	JJΔupt was transformed with p002-253 and pPmcr_JJ-uidA, plasmid was removed afterwards.	This study
JmcrRJ	*Δupt Δacs(107-1075)::*P_{mcrR_JJ}*-uidA*	JJΔupt was transformed with p002-253 and pPmcrR_JJ-uidA, plasmid was removed afterwards.	This study
JflaJ	*Δupt Δacs(107-1075)::*P_{fla_JJ}*-uidA*	JJΔupt was transformed with p002-253 and pPfla_JJ-uidA, plasmid was removed afterwards.	This study
JnifΔnrpR	*Δupt ΔnrpR Δacs(107-1075)::*P_{nif}*-uidA*	Jnif was transformed with p002-247 and pΔnrpR	This study

Table S3. Plasmid maps

Plasmid	link
pMM002P	https://benchling.com/s/seq-NcNgENuHGpjcjuVSBU0e?m=slm-uWqdWXhT0CNxlHaCyTYG
pMM005	https://benchling.com/s/seq-hvquLQ7RbCe0LrHLvgUG?m=slm-Uljuux0W4UWZCD8H0UbE
p002-218	https://benchling.com/s/seq-95SMUqzI7CM8HY637Tej?m=slm-nJXjd3LjQAAQGNstiyv0
p002-226	https://benchling.com/s/seq-lsoPOqfmcoajKn9cMy8o?m=slm-KeQfniz32M9QIHnTm6ob
p002-218-L1000	https://benchling.com/s/seq-OzWgU1lZMoUM48r8Jnli?m=slm-4wjgalWGU6q2jfQJBPjy
pPmtr-uidA	https://benchling.com/s/seq-0Gomnk1heQqOZcCU6GRn?m=slm-dHj8kpnVmpgyZ1LyXbNf
pPmcr-uidA	https://benchling.com/s/seq-W11SbMBtaTdab5nlcUC9?m=slm-gmg7fHunIevaxHSKpRLf
pPmcrR-uidA	https://benchling.com/s/seq-zpHKgTXargZcOH0sg26M?m=slm-b8KraXsjOZP1H77qMZ2N
pPhdrC1-uidA	https://benchling.com/s/seq-7vfw1HM5KaJn0JQZxqKr?m=slm-ACTIrP1pfkZRr5MR2y0T
pPhdrC2-uidA	https://benchling.com/s/seq-GECSSScdMhnf9XM0i7dH?m=slm-bF0EK8QlLhtSH4lvUW21
pPhdrA2-uidA	https://benchling.com/s/seq-Jwx0H7YJcMCzA42eJPF6?m=slm-cwKCm0LKbQLv1cN4IlaC
pPfdh-uidA	https://benchling.com/s/seq-DOMwbRZ6WALbE3SHTTMF?m=slm-sUQWlj4qbdmmL74pSb1Q
pPeha-uidA	https://benchling.com/s/seq-vPCMYrXjr9PiLKxHatOs?m=slm-UbqyKGuyakFfSQ4STlse
pPeha w.TF-uidA	https://benchling.com/s/seq-TtXDra2oDlyADm8eS7ns?m=slm-CEUoenlUGVfMb8BPeabC
pPehb-uidA	https://benchling.com/s/seq-tDXok4O6Ric798CaMapy?m=slm-Hd9WyxMjMk7omegb0obG
pPglnA-uidA	https://benchling.com/s/seq-RcddggPTElJMqlZ2s6Eh?m=slm-JKc8i5HNtjdRbuw51yn1
pPnif-uidA	https://benchling.com/s/seq-iaa8yge2Lqaj3XpOCt0Y?m=slm-e7qq4iYbyZtirETKuQt0
pPmcr_JJ-uidA	https://benchling.com/s/seq-6sP4BHn8cSssampgokXB?m=slm-GELjQGAhiabG2JUlaKCl
pPmcrR_JJ-uidA	https://benchling.com/s/seq-3wN9rk0VvzFd5cZO1UxY?m=slm-aHvtqEanTqm85apTBcJm
pPfla_JJ-uidA	https://benchling.com/s/seq-D6xKkW9YqDEBIvTbz046?m=slm-68Z2g0Tdg8d9wlolLIAX
pΔnrpR	https://benchling.com/s/seq-ZaGqJIBsQxrGJb9RARmb?m=slm-APrpwwhfkbpBKSUMw7Qs

Table S4. Primers used for pMM002P construction

Fragment	Subfragment	Primer	Sequence (5'->3')	Template
F1		P1	CTTCTTCAGGGAGCTCGAGATAAGAATTACTAGATCGGAAAATTCAGTAATGCAAAAACAC	*Methanococcus voltae* A3
		P2	TTTAATTAAGTATTTTAATTATTCTTTTTTCATTATTTTATTAAGTTATTAC	
F2	F2.1	P3	GTAATAACTTAATAAAATAATGAAAAAAGAATAATTAAAATACTTAATTAAATTAGCTGGTCTGGGCGTACTC	pY016
		P4	GCCCTGATGAGCCTGATGCTTCAGATGCGGAACAGCATCAC	
	F2.2	P5	GTGATGCTGTTCCGCATCTGAAGCATCAGGCTCATCAGGGC	pY016
		P6	GTGAAGGTGGAGAAGCAGGTTTATCAGAAGTTCGAGAAGATGCTG	
	F2.3	P7	CAGCATCTTCTCGAACTTCTGATAAACCTGCTTCTCCACCTTCAC	pY016
		P8	GGATAAGAAGTACGCCAAGTGCTTACAGAAGATCGACAAGGACGATG	
	F2.4	P9	CATCGTCCTTGTCGATCTTCTGTAAGCACTTGGCGTACTTCTTATCC	pY016
		P10	CCTTCAAGAAGATCGGCTCCTTTTCTTTAGAGCAGTTACAGGAGTACGCCGACGCCG	
	F2.5	P11	CGGCGTCGGCGTACTCCTGTAACTGCTCTAAAGAAAAGGAGCCGATCTTCTTGAAGG	pY016
		P12	GTTTAAGCCACTGTATAAGCAGGTTCTGAGCGATCGGGAGTCTCTG	
	F2.6	P13	CAGAGACTCCCGATCGCTCAGAACCTGCTTATACAGTGGCTTAAAC	pY016
		P14	ATATATATACCTATATAGTGTCGAGGTGTAATAATATGAGCAAGCTGGAGAAGTTTAC	
F3	F3.1	P15	ATTATTACACCTCGACACTATATAGGTATATATAT	*Methanococcus voltae* A3
		P16	GTTATATTTTGATCGATCAGCTGAATTAACGCCGGCGAATTTTATTAAATTATCATATAACTATATAAATTTATC	
	F3.2	P17	AATTCGCCGGCGTTAATTCAGCTGATCGATCAAAATATAAC	pMEV4
		P18	CAATGCAGGTGCGGATCCTCACCTGCGCATATCTACACTTAGTAGAAATTCACTCTAGTATTAG	
	F3.3	P19	ATGCGCAGGTGAGGATCCGCACCTGCATTGGATATAGCAAAAGTGGGACTTAAGTTCC	pMEV4mTs
		P20	GGAAGGTCGTCTCCTCTAGT	
	F3.4	P21	AAACTAGAGGAGACGACCTTCCATGATAAAAGATGAAAGATGGGAAGGAG	*Methancoccus maripaludis* S2
		P22	GTTGGTTTATATTCTGTCATGCATTTCACCTATTTTATTCTAAATTGTGCATTTGTGGGAGC	
	F3.5	P23	AATAGGTGAAATGCATGACAGAATATAAACCAACAGTTAGATTAGC	pMEV4
		P24	CGGCTTCGGTCGGAGCCATGGTTATGCTCCTGGTTTTCTTGTCATAC	

The fragment F1, subfragments F2.1 to F2.6, and F3.1 to F3.5 were amplified by normal PCR. The fragments F2 and F3 were constructed by jointing F2.1 to F2.6 and F3.1 to F3.5 together by overlap PCR. pMM002P was constructed by assembling NcoI/BcuI digested pLW40, F1, F2, F3 by Gibson cloning[8].

Table S5. Primers used for pMM005 construction

Fragment	Subfragment	Primer	Sequence (5'->3')	Template
F1		P1	CTTCTTCAGGGAGCTCGAGATAAGAATTACTAGATCGGAAAATTCAGTAATGCAAAAACAC	*Methanococcus voltae* A3
		P2	TTTAATTAAGTATTTTAATTATTCTTTTTTCATTATTTTATTAAGTTATTAC	
F4	F4.1	P25	GTAATAACTTAATAAAATAATGAAAAAAGAATAATTAAAATACTTAATTAAATTAGTCGCCTCCCAGCTGAG	pX165
		P26	GAGAATGCTGGCCTCTGCCGGCGAACTTCAGAAGGGAAACGAACTGG	
	F4.2	P27	AGGGCCAGTTCGTTTCCCTTCTGAAGTTCGCCGGCAGAGGCC	pX165
		P28	GAAAACACCCAGCTTCAGAACGAGAAGCTGTACCTGTACTACCTTCAGAATGGGCGGGATATGTAC	
	F4.3	P29	CTGAAGGTAGTACAGGTACAGCTTCTCGTTCTGAAGCTGGGTGTTTTCCACGG	pX165
		P30	AGCCCCGCCATTAAGAAGGGCATCCTTCAGACAGTGAAGGTGGTGG	
	F4.4	P31	CTCGTCCACCACCTTCACTGTCTGAAGGATGCCCTTCTTAATGGC	pX165
		P32	ACCTGGCCGAGGATGCCAAACTTCAGCTGAGCAAGGACACCTAC	
	F4.5	P33	GTCGTCGTAGGTGTCCTTGCTCAGCTGAAGTTTGGCATCCTCGGCCAG	pX165
		P34	CATCAATCCATATATTATATATATACCTATATAGTGTCGAGGTGTAATAATATGGACAAGAAGTACAGCATCG	
F5	F3.1	P15	ATTATTACACCTCGACACTATATAGGTATATATAT	*Methanococcus voltae* A3
		P16	GTTATATTTTGATCGATCAGCTGAATTAACGCCGGCGAATTTTATTAAATTATCATATAACTATATAAATTTATC	
	F5.1	P17	AATTCGCCGGCGTTAATTCAGCTGATCGATCAAAATATAAC	pMEV4
		P35	TGATAACGGACTAGCCTTATTTTAACTTGCTATTTCTAGCTCTAAAACCAATGCAGGTGCGGATCCTCACCTGCGCATCACTCTAGTATTAGTTATCTATAAAATTATAATATCAATAGC	
	F5.2	P36	AGCAAGTTAAAATAAGGCTAGTCCGTTATCAACTTGAAAAAGTGGCACCGAGTCGGTGCTTTTGATATAGCAAAAGTGGGACTTAAGTTCCC	pMEV4mTs
		P20	GGAAGGTCGTCTCCTCTAGT	
	F3.4	P21	AAACTAGAGGAGACGACCTTCCATGATAAAAGATGAAAGATGGGAAGGAG	*Methancoccus maripaludis* S2
		P22	GTTGGTTTATATTCTGTCATGCATTTCACCTATTTTATTCTAAATTGTGCATTTGTGGGAGC	
	F3.5	P23	AATAGGTGAAATGCATGACAGAATATAAACCAACAGTTAGATTAGC	pMEV4
		P24	CGGCTTCGGTCGGAGCCATGGTTATGCTCCTGGTTTTCTTGTCATAC	

The subfragments F4.1 to F4.5, and F5.1 to F5.2 were amplified by normal PCR. The fragments F4 and F5 were constructed by jointing F4.1 to F4.5 and F3.1, F5.1, F5.2, F3.4 and F3.5 together by overlap PCR. pMM005 was constructed by assembling NcoI/BcuI digested pLW40, F1, F4, F5 by Gibson cloning[8].

Table S6. The rest of primers used in this study

Primer	Sequence (5'->3')	
CR227	GCTTATAATGTTTTTAATTTTTAAAAACAGATAAATTTATATAGTTATATGATAATTTAATAAAATTCGCCCAAATTTTGTAATGAATATGTGGAGTCTTTCATTT	L1000
CR228	GTACAATGTCTGCTCAGACTGCGCGGCCGCCTGCGTCAGGAAAAACTACTTC	
CR229	GAAGTAGTTTTTCCTGACGCAGGCGGCCGCGCAGTCTGAGCAGACATTGTAC	
CR230	GTTATTTATGTTATATTTTGATCGATCAGCTGAATTAACGCCGGCAACTTATTCTTTCGGGGGAAATC	
CR231	GCTTATAATGTTTTTAATTTTTAAAAACAGATAAATTTATATAGTTATATGATAATTTAATAAAATTCGCCGACCGCTGTCTATTGAATAAGC	D250
CR232	GAATGGGAAGATGAAGGGGACGCGGCCGCGGTCTGTCGAAGGAGCTGTTG	
CR233	CAACAGCTCCTTCGACAGACCGCGGCCGCGTCCCCTTCATCTTCCCATTC	
CR234	GTTATTTATGTTATATTTTGATCGATCAGCTGAATTAACGCCGGGCATACAAGAGCATTTTATGAAAAAGATATTG	
CR235	GCTTATAATGTTTTTAATTTTTAAAAACAGATAAATTTATATAGTTATATGATAATTTAATAAAATTCGCCTTTATCGCATAGACCAAAAGTACTTC	D500
CR236	CATGGTTGCAAAAGTATCAAGTCAGCGGCCGCCATTACAAGAGGTGTTTTTGAATGG	
CR237	CCATTCAAAAACACCTCTTGTAATGGCGGCCGCTGACTTGATACTTTTGCAACCATG	
CR238	GTTATTTATGTTATATTTTGATCGATCAGCTGAATTAACGCCGGTCTTAATTGAAGGAGAAGAGTCATCC	
CR239	GCTTATAATGTTTTTAATTTTTAAAAACAGATAAATTTATATAGTTATATGATAATTTAATAAAATTCGCCCCATTCAAAAACACCTCTTGTAATGAC	L500 (amplified with CR228 and CR229)
CR240	GTTATTTATGTTATATTTTGATCGATCAGCTGAATTAACGCCGGTTTGACATGGTTGCAAAAGTATCAAG	
CR241	GCTTATAATGTTTTTAATTTTTAAAAACAGATAAATTTATATAGTTATATGATAATTTAATAAAATTCGCCAAAGCAACAGCTCCTTCGAC	L250 (amplified with CR228 and CR229)
CR242	GTTATTTATGTTATATTTTGATCGATCAGCTGAATTAACGCCGGCAAACATTGAATGGGAAGATGAAG	
CR243	GCTTATAATGTTTTTAATTTTTAAAAACAGATAAATTTATATAGTTATATGATAATTTAATAAAATTCGCCATTTTAGACGTATTTTCGTCGAGTTC	P_{fla_JJ}-*uidA*-T_{fla}
CR244	GGGGTTTCTACAGGACGTAACATGATTGAACCTCCTAAGTTCACCAG	
CR245	CAGCAGGGAGGCAAACAATGATTTATTTAAAAAATCATTTGAATAGTGAACCG	
CR246	GTTATTTATGTTATATTTTGATCGATCAGCTGAATTAACGCCGGGCAGTTCCAAATTTCAACATTTTATCAC	
E275	ATGTTACGTCCTGTAGAAACCCC	
CR31	TCATTGTTTGCCTCCCTGCTG	
CR394	GGAGAGCAGGCAAAATGTTTAGCTTTTTACCACCTGAAAGAAAAACTTATAC	P_{nif}
CR395	GGGGTTTCTACAGGACGTAACATACTAAAGCCTCTATTGCATCATC	
CR396	GGAGAGCAGGCAAAATGTTTAGTCCTATGAATATTTAAGGAGGCCTTTTGG	P_{mtr}
CR398	GGGGTTTCTACAGGACGTAACATTATTTCACCTCACAATATACGGGAATG	
CR399	GGAGAGCAGGCAAAATGTTTAGATTACCATCTTATTTACTTTTATAAAAATAGTAGTAAGTAG	P_{mcr}
CR400	GGGGTTTCTACAGGACGTAACATAGGAACCACTCCTATTTTTTTGATATATACATC	
CR401	GGAGAGCAGGCAAAATGTTTAGAGGAACCACTCCTATTTTTTTGATATATAC	P_{mcrR}
CR402	GGGGTTTCTACAGGACGTAACATATTACCATCTTATTTACTTTTATAAAAATAGTAGTAAGTAG	

CR403	GGAGAGCAGGCAAAATGTTTAGAAGAGCATAGTTTATATCGTTTATACCATC	P_{hdrC2}
CR404	GGGGTTTCTACAGGACGTAACATCAGTAACACCTCGAGGATACTTTTG	
CR405	GGAGAGCAGGCAAAATGTTTAGGCACCCGATTTCAACAACC	P_{hdrC1}
CR406	GGGGTTTCTACAGGACGTAACATTCGCTTCACCTCCCTTATAGTTTAATTTTTC	
CR407	GGAGAGCAGGCAAAATGTTTAGGAAATCCCTCGCACATAGAGAAC	P_{hdrA2}
CR408	GGGGTTTCTACAGGACGTAACATGGATTCACCTCCACATATAACAAGTAAG	
CR411	GGAGAGCAGGCAAAATGTTTAGCTGTGAAGAAAGAAACGTTATAGATGG	P_{fdh}
CR412	GGGGTTTCTACAGGACGTAACATTAATCATCACCGTTAAATTATGTGTGAC	
CR413	GGAGAGCAGGCAAAATGTTTAGAAAAAATCACCTTAATCAAATTTTGAAAATGC	P_{eha}
CR414	GGGGTTTCTACAGGACGTAACATTTTACCACGATTTAGTCTACAAGACTTTAAATTATTTC	
CR414L	GGGGTTTCTACAGGACGTAACATTAATATCACCAGCAGTGTACAATCATAC	$P_{eha\ w.TF}$ (amplified with CR413)
CR419	GGAGAGCAGGCAAAATGTTTAGGTCTATCACACAAAAGGGATTTTTGTAATG	P_{ehb}
CR420	GGGGTTTCTACAGGACGTAACATATTACCACCAATCGGGACTAAAAATTTG	
CR421	GGAGAGCAGGCAAAATGTTTAGTAAATCACCGTGAATTTGCGAATAATTTC	P_{glnA}
CR422	GGGGTTTCTACAGGACGTAACATATATATTCCTCCAAAATCATCTTTATAGGAAATAGC	
CR467	GGAGAGCAGGCAAAATGTTTAGTTTATAGATTCCCGATGTTCAAATGC	P_{fla_JJ} (amplified with CR244)
CR468	GGAGAGCAGGCAAAATGTTTAGATTACCATCTTATTTAGCTTTATAAAAATAGTAATC	P_{mcr_JJ} (amplified with CR400)
CR469	GGGGTTTCTACAGGACGTAACATATTACCATCTTATTTAGCTTTATAAAAATAGTAATC	P_{mcrR_JJ} (amplified with CR401)
CR416	CAAAAGTGGGACTTAAGTTCCCACTTTTGCTATTGATTTATCAACATTGAGGCTTTTAGG	For suicide plasmid construction incl. homologous arms, *uidA* and vector
CR417	GTGCACCAAGGATCTTCACCTAGAACTAGTGTAGATGTTCTAAAAGATAATGATTATTTAGTATTTG	
CR418	GTTCCTGGCCTTTTGCTGGCCAAGCTTATTGTAAATTATGCCTCCAAGGGTG	
CR298	CTAAACATTTTGCCTGCTCTCC	
CR415	GGGAACTTAAGTCCCACTTTTGCTATATCTCATTGTTTGCCTCCCTGCTG	
SV-F	TCTAGGTGAAGATCCTTGGTGCAC	
SV-R	GGCCAGCAAAAGGCCAGGAAC	
CR426	GTTCCTGGCCTTTTGCTGGCCAAGCTTGGATTTACCTGTTGTTATGTATTCATTTACC	ΔNrpR
CR427	GTATATTACGAATAGGGCGTTTTTTATTTATAAAATGGATAAAATTTCAACATCAATATTACTGTC	
CR428	GAAATTTTATCCATTTTATAAATAAAAAACGCCCTATTCGTAATATACTATTC	
CR429	GGAAGGTCGTCTCCTCTAGTTTTTTTTGATATATACATCATAACATTACTCTATG	
CR430	ACTAGAGGAGACGACCTTCCATGGGAAAAAAGGACAAAAGATGG	
CR431	GTGCACCAAGGATCTTCACCTAGAACTAGTCATTTCAGTTTACGGAAAAACTGTTTC	

Supplementary Methods

Additional details for utilizing the CRISPR/Cas genome-editing toolbox in *M. maripaludis* are provided below:

CRISPR/Cas plasmids and their functional elements. CRISPR/Cas plasmids are designed and constructed to contain expression cassettes for gRNAs and Cas proteins, either *Lachnospiraceae bacterium* (Lb) Cas12a as in pMM002 or *Streptococcus pyogenes* (Sp) Cas9 as in pMM005. Both of the CRISPR/Cas plasmids also contain the following elements: ampicillin (*amp*) and puromycin (*pac*) resistance genes for selection in *E. coli* and *M. maripaludis*, respectively, a suicide gene (*upt*) for rapid removal of plasmids from *M. maripaludis*, and respective ori sequences for autonomous replication in either *E. coli* or *M. maripaludis*. The gRNA included is made up of a fixed sequence that will be bound by the Cas protein, plus a non-sense sequence that is meant to be substituted according to the genomic DNA sequence that is going to be targeted. Upon digestion with *PaqC*I, the non-sense sequence from this gRNA is removed, and two sticky ends are created. Thus, the genome-targeting gRNA sequence that is to be designed must be flanked by these sticky ends as well.

Design of gRNA sequences via the CHOPCHOP webtool. Obtaining genome-targeting gRNA sequence necessitates designing two single-stranded oligonucleotide primers, which is accomplished by following the CHOPCHOP step-by-step instructions (https://chopchop.cbu.uib.no/). For this, the gene name (including RefSeq and ENSEMBL gene IDs) or the pasted nucleotide sequence of interest is entered as the target and the *M. maripaludis* JJ (DSM 2067) strain is selected as the reference genome. The type of Cas protein (CRISPR mode) is then selected, and the advanced options display is opened. For LbCas12a (Cpf1), 20-24 and TTTN are entered for the sgRNA length without PAM and the 5'-PAM, respectively. For SpCas9, 20 and NGG are entered for the sgRNA length without PAM and the PAM-3', respectively. CHOPCHOP output will list several suitable gRNA sequences for CRISPR editing within the nucleotide sequence of interest. It is recommended to select three of the top 10 gRNA sequences. Two oligonucleotide primers are designed based on these sequences without the 5'-PAM. The forward primer consists of the complementary nucleotide sequence of the forward cohesive end site (established by *Paq*CI digestion on pMM002P or pMM005), followed by the nucleotide sequence provided by CHOPCHOP. The reverse primer is fashioned in a similar manner, consisting of the reverse complementary sequence of the reverse cohesive end site (established by *Paq*CI digestion on pMM002P or pMM005), followed by the reverse complementary sequence of the nucleotide sequence provided by CHOPCHOP. Forward and reverse primers are annealed, with the resulting double-stranded DNA fragment then ligated into the plasmid (pMM002P or pMM005) backbone via the *Paq*CI cohesive ends. Provided below are examples of the above-mentioned components.

Nucleotide sequence of gRNA with the 5'-PAM underlined:

<u>TTTN</u>AAAATTTTCCCCGGGGAAAA

Forward oligonucleotide primer:

5'-AGATAAAATTTTCCCCGGGGAAAA

Reverse oligonucleotide primer:

5'-TATCTTTTCCCCGGGGAAAATTTT

5'- and 3'-overhanging *Paq*CI cohesive ends in the plasmid backbone:

5'- ····NNNN // GATANNNN····
3'- ····NNNNTCTA // NNNN····

Repair fragment (RF) insertion. The RF is introduced into *M. maripaludis* cells separately as a suicide plasmid or PCR fragment, or else by being included within the CRISPR/Cas plasmid. For the latter option, the presence of an *Mre*I restriction site between the gRNA and Cas elements will facilitate RF insertion.

Quick natural transformation protocol[7] in *M. maripaludis*:

1. Grow a 5 mL of *M. maripaludis* culture in McC medium until OD between 0.7 and 1.2 is reached. The freshness of the seed culture used to inoculate the culture to be transformed positively affects the transformation efficiency.
2. Once the desired OD is reached, add 2 μg of the CRISPR plasmid (plus 2 μg of the linear repair fragment or the suicide plasmid, if provided separately).
3. Flush the culture with 80% H_2, 20% CO_2, for 0.5 min, and pressurize the culture to 2.8 bar (absolute pressure).
4. Incubate the culture on a shaker at 37°C, 200 rpm, for 4 hours.
5. Plate an appropriate amount on McC medium with puromycin.

Promoter sequences used in this study

P_{mtr} from *Methanococcus vannielii* SB

tcctatgaatatttaaggaggccttttggcttcctttttttatacctattatatattgtttcggaatcattaacaatattgctaattaaaatata
ctttaattaaattaaaggaaaattaggctttgtttttttaaaaaaacgaatgcaaagtaatatatatagtataacaagatattaactgatta
gctaacggcatagataacgttctaggctttaaagccactagaagtcatagctaacattcccgtatattgtgaggtgaaata

P_{mcr} from *Methanococcus vannielii* SB

attaccatcttatttacttttataaaaatagtagtaagtagtaattcattaaaaatagaattccaatattagtttttgtcatgctaattacaa
atttatctagtattacgtagcatgataagatataattaacctaatccaaaacttaatccccgatagagactatatatatttttctatttgatt
ttaatgaaaacttgaatatatcttcctttaataatgttatgatgtatatatcaaaaaaataggagtggttcct

P_{mcrR} from *Methanococcus vannielii* SB

aggaaccactcctatttttttgatatatacatcataacattattaaaggaagatatattcaagttttcattaaaatcaaatagaaaaatat
atatagtctctatcggggattaagttttggattaggttaattatatcttatcatgctacgtaatactagataaatttgtaattagcatgaca
aaaactaatattggaattctatttttaatgaattactacttactactatttttataaaagtaaataagatggtaat

P_{eha} from *Methanococcus vannielii* SB

aaaaaatcaccttaatcaaattttgaaaatgctacttacctaagtaaaagactatgttctcataacataaaaaaatttatacatatcttaa
ttttcgacttgaaccaagtaataaaatatatatacaaaatccctcaagaataatgttattcttatgaaataatttaaagtcttgtagacta
aatcgtggtaaa

$P_{eha\ w.\ TF}$ from *Methanococcus vannielii* SB

Aaaaaatcaccttaatcaaattttgaaaatgctacttacctaagtaaaagactatgttctcataacataaaaaaatttatacatatctta
attttcgacttgaaccaagtaataaaatatatatacaaaatccctcaagaataatgttattcttatgaaataatttaaagtcttgtagact
aaatcgtggtaaaatgtacgacaaattaaagattattgaaagggcaattcttttaaacccggaacatatcaaggtttttcgtgaaaagct
caaaattacccagtcaaaacttgctgaagaaagtggtgtaagtcaatcacacctaagtatgcttgaaaagggtaaacgggatactgga
gaagctcatgctgccgcaataacacttggaatgcttaaatgtagtaatgcatgggataaagaagacccaatactatatcttttagacgtt
ttatcgcttacaaagttagaaagcgccattgtatcgtttataagagatttgactacaacaaacagtattctttacaggagatttattgaag
ggcatcctgtatatattgtagatataaaccaattttctgaagaaattaaaaagagactaaaagtaatgaatatcgaagaagtatcctttt
ttaggggccggatggaaattaaaggaatacatttggataataaaacagtagtaataagtttggattgctcagatattcgtagacttgaa
aaaaaggtctctgatgtactttcaaataatacgataatacagatatttccaaaagacgaaattcccccaatatattctattaaaaaagaa
tgtatgattgtacactgctggtgatatta

The coding sequence of the putative transcription factor is underlined.

P_{ehb} from *Methanococcus vannielii* SB

gtctatcacacaaaagggatttttgtaatgccaaaaaattgaatatgccaagaataatggcgtataaaaatataaaactgatgttctaa
atctttaaccctatttttctaataatttttaaaactctttttaacgtttaatctaaaaatggggggttttatgccatttaacgaataaatacg
cctatcaatatactattaatatataaacgaggtgaaaaaataatggcagacgaaaaaagaagggctgaacaaaagaaaaaagaaag
ttaccaaaaacaggctaatcaggaacgacaatcaaaacagagaaagagtgaagaattaaagaaaaaagaatgggcccacaaaaag
taggagttattatcttatttttaccatatttctatttttaaattatttcataaaaaaaaggcatccttaaatgatttggcattattttgagtttt
agtagatttttcaagtaccataacttatttatatctagtttttaaaggtatttatcattataattaatgatagattaagaggaattcttagtt
agtgtctttgatccaaatttttagtcccgattggtggtaat

P_{hdrA2} from *Methanococcus vannielii* SB

gaaatccctcgcacatagagaactgactcttaaaatagtatttcgtacagtatttatagattactaaataaaaagtttttatttttaaagtt
tagaattttaaactaataacaaaagaataataaatttttttaataattttaaaataaaaaaagaaataaacgtttagaggggtaagaaat
agggggtggttttatgttatgtaaagtatgatttactctaaaaatagaacggggaggggttatgttctaatttttataattttaatgtttttg
taaattgttaaaacttattataacttgttaaaggtaattaaaagcgtatttttcatatgaggggtgggaatgggattaggagatataaatc
tcctacacggtgttatcagtagtgaagtgggggtatgaggggggtttaaaataataatctcactactgtcccacaccgtgcaaagtatac
cttcaaacttaatatatttaaactttatggttgcttaatcaatgactttttttaaaatattattcaataacgattatacttattttaatattatat
attattacaaagacattacatcgcctaattaatattgttaattgcccatataatacaaatctttatgggggttttagaattatagaaaatat
ttttttattattgaattaattagttttaggccccatattataatatctaatattaatgggcagtattaggaatttcaagaatgtatctttttata
atgaggtgagtgtagtataaatgtataccaattaccttacttgttatatgtggaggtgaatcc

P$_{hdrC2}$ from *Methanococcus vannielii* SB

aagagcatagtttatatcgtttataccatcttccatttttaccacgattagttaattttggattagactgatttaatttccctttttaaaatgtt
tacaacatattcttttgtatcacatgatataagtactgttttttggggcgaagttgttactggatttagttcaacatctgttttaaaaatttct
tttaaaaattttaaaatggctttatttgcccttccttcaattggttgttctttaatttttacttcaagtcttttacgccatttattaatttttccaa
tttcgttctttttagcattagtagtaacctctatatcgattaaaactcctttttcagtttttttgattatttcttcaatcaaaataccaccttctt
attactattttgaaacaaggtaaaaataaattattatattatacaacagtataatatagggcagcaactaataataaatatattatatcc
gcacaaaagtatcctcgaggtgttactg

P$_{hdrC1}$ from *Methanococcus vannielii* SB

gcacccgatttcaacaacccttgcagcactccaaaggtgaatgtgtgaacaaattccgcatattttttcgcacaatatacatgctttttca
acgggaagtccctccattaacctctctacacccctatggtttacaccaattgtaagttcagcatcttttactatttcatcttcaataaaaagt
cttaaacgatgcggttcaagcattgttgcgtgaacaggcccaattgcgatttctccttcgtacatgattctcacttttaaagtaatttataat
aatctaactcgattgaaattatacgtaagtatatagaaaactaatatttatcgttattaattaatcatcattattgattatgataattatag
attagtaatccccttttatattattaattattatccaataccctattatatacctatataataggtgaataaattagtcaaatatatatccag
tttcaaagaaaaattaaactataagggaggtgaagcga

P$_{fdh}$ from *Methanococcus vannielii* SB

ctgtgaagaaagaaacgttatagatggtgtaggacttctaaaggaacatcctgcaatacccaaagatgtcgcaattgatgggtatctta
tgaatattgaatcgggaaagttaattaatatagcctaaataactgtggatgatgcccaaaggggcggacacctccgtatctaaccccat
agagtcaagccaactcaagtattagccaaatagttcagataaactagcctaactttatctgcctacactaaatactcaaaatacatttat
catttttcatattttaatttattctttttattatttttttatattaagtactgataaagtaatatagcacgatattattttttatattaagtactgat
aaagtaatatagcacgaaaaattgaaaatagcacacaataattatagcacaaatggattagtatgaactaatttggaatactaatcttt
aactgaaaaccaaaaggtgtcacacataatttaacggtgatgatta

P$_{mcr\_JJ}$ from *Methanococcus maripaludis* JJ

attaccatcttatttagctttataaaaatagtaatcaataataatctattaaaaacatccgttttatctcaacaatagcagggttaatttcg
atttaacacagtattacgtactaaaaatcgagatatttaacctaatctaaaacttaatcgatgatggagagtatatatatttttctatttga
gtttaatgaaaacttgaatatatattctttcagtaatgttatgatgtatatatcaaaaaaataggagtggttcct

P$_{mcrR\_JJ}$ from *Methanococcus maripaludis* JJ

aggaaccactcctatttttttgatatatacatcataacattactgaaagaatatatattcaagttttcattaaactcaaatagaaaaatat
atatactctccatcatcgattaagttttagattaggttaaatatctcgatttttagtacgtaatactgtgttaaatcgaaattaaccctgcta
ttgttgagataaaacggatgttttaatagattattattgattactatttttataaagctaaataagatggtaat

P$_{fla\_JJ}$ from *Methanococcus maripaludis* JJ

tttatagattcccgatgttcaaatgcatattttccaacagaaagaacatctcgaaaatctgaaaaatcaaaaccagatattccttttgtat
ctgcaccgctttttggatttaaatgataatcagttgatttaaaagatcctatcattattatcacgcttttaatagtaatattattatgaattaa

atggattatattcgtttttttgcatgtgtttgtgtaggttaaccgtttttttcattaaatttacctacataatcatatacataactacatacaaaa
ccaaatacagcaccatatgccaaaataagtgcaaaaaaagtatatattaacaaaaaacgacatatttcttcgggaatatccccgaaga
ggtaaaaatctcaaggattcatacgagattctaaaggctagaaactctcgaaaagaaagtctggtgaacttaggaggttcaatc

BRE region is in red; TATA box is in blue; ribosome binding site is in green; transcription factor EarA binding sites are in orange; transcription start site is in gray box[9].

P_{glnA} from *Methanococcus vannielii* SB

taaatcaccgtgaatttgcgaataatttcataattatctgaaaagtatatcaaactatccaaatattaaaaattaccgccatttaaaatat
attgaatgtaccgtaaactatatatattgaaaaagcggaaagctatttcctataaagatgattttggaggaatatat

Two putative TATA boxes are in blue. The two putative TATA box are overlapped. The first TATA box is underlined, and the second TATA box is in yellow box; operator is in purple; ribosome binding site is in green; putative transcription start sites are in gray box[10].

P_{nif} from *Methanococcus vannielii* SB

ctttttaccacctgaaagaaaaacttatacttaatatattttcttagtaatttaaagaagtttcgtattgaattaaataaaaagttaaaatc
cacagttaacttttatgtaatctttattttatgattaatgttattttattagctaatccgaaagatttatatattggaatatagtatatgatatt
tcaccggaaagttacttccgttaaataataaatataaggaattaaaattcctaaaatgatgatgcaatagaggctttagt

TATA box is in blue; transcription start site is in gray box; operators are in purple[1].

Reference

(1) Dodsworth, J. A.; Leigh, J. A. Regulation of nitrogenase by 2-oxoglutarate-reversible, direct binding of a PII-like nitrogen sensor protein to dinitrogenase. *Proc. Natl. Acad. Sci. U. S. A.* **2006**, *103* (26), 9779–9784. https://doi.org/10.1073/pnas.0602278103.

(2) Walters, A. D.; Smith, S. E.; Chong, J. P. J. Shuttle vector system for *Methanococcus maripaludis* with improved transformation efficiency. *Appl. Environ. Microbiol.* **2011**, *77* (7), 2549–2551. https://doi.org/10.1128/AEM.02919-10.

(3) Lyu, Z.; Jain, R.; Smith, P.; Fetchko, T.; Yan, Y.; Whitman, W. B. Engineering the autotroph *Methanococcus maripaludis* for geraniol production. *ACS Synth. Biol.* **2016**, *5* (7), 577–581. https://doi.org/10.1021/acssynbio.5b00267.

(4) Zetsche, B.; Gootenberg, J. S.; Abudayyeh, O. O.; Slaymaker, I. M.; Makarova, K. S.; Essletzbichler, P.; Volz, S. E.; Joung, J.; van der Oost, J.; Regev, A.; Koonin, E. V.; Zhang, F. Cpf1 Is a single RNA-guided endonuclease of a Class 2 CRISPR-Cas system. *Cell* **2015**, *163* (3), 759–771. https://doi.org/10.1016/J.CELL.2015.09.038.

(5) Ran, F. A.; Hsu, P. D.; Wright, J.; Agarwala, V.; Scott, D. A.; Zhang, F. Genome engineering using the CRISPR-Cas9 system. *Nat. Protoc.* **2013**, *8* (11), 2281–2308. https://doi.org/10.1038/nprot.2013.143.

(6) B.Whitman, W.; Shieh, J.; Sohn, S.; Caras, D. S.; Premachandran, U. Isolation and characterization of 22 mesophilic *Methanococci*. *Syst. Appl. Microbiol.* **1986**, *7* (2–3), 235–240.

(7) Fonseca, D. R.; Halim, M. F. A.; Holten, M. P.; Costa, K. C. Type IV-like pili facilitate transformation in naturally competent archaea. *J. Bacteriol.* **2020**, *202* (21), 1–12. https://doi.org/10.1128/JB.00355-20.

(8) Gibson, D. G.; Young, L.; Chuang, R. Y.; Venter, J. C.; Hutchison, C. A.; Smith, H. O. Enzymatic assembly of DNA molecules up to several hundred kilobases. *Nat. Methods* **2009**, *6* (5), 343–345. https://doi.org/10.1038/nmeth.1318**.**

(9) Ding, Y.; Berezuk, A.; Khursigara, C. M.; Jarrell, K. F.; Maupin-furlow, J. A. Bypassing the Need for the Transcriptional Activator EarA through a Spontaneous Deletion in the BRE Portion of the Fla Operon Promoter in *Methanococcus Maripaludis. Fronters in Microbiology*. **2017**, *8*, 1–10. https://doi.org/10.3389/fmicb.2017.01329.

(10) Cohen-kupiec, R.; Marx, C. J.; Leigh, J. A. Function and Regulation of GlnA in the Methanogenic Archaeon *Methanococcus Maripaludis*. *J. Bacteriol.* **1999**, *181* (1), 256–261.
